# Supplementary material for: Characterization of Novel Human β-glucocerebrosidase Antibodies for Parkinson Disease Research
Source: bioRxiv. 2023 Sep 15:2023.09.14.557851. Preprint. [Version 1] doi: 10.1101/2023.09.14.557851 (PMC10602026; doi:10.1101/2023.09.14.557851)
Supplement: 1 [file NIHPP2023.09.14.557851V1-supplement-1.pdf]

bioRxiv preprint doi: https://doi.org/10.1101/2023.09.14.557851; this version posted September 15, 2023. The copyright holder for this preprint (which was not certified by peer review) is the author/funder. This article is a US Government work. It is not subject to copyright under 17 USC 105 and is also made available for use under a CC0 license.

Supplementary Figures

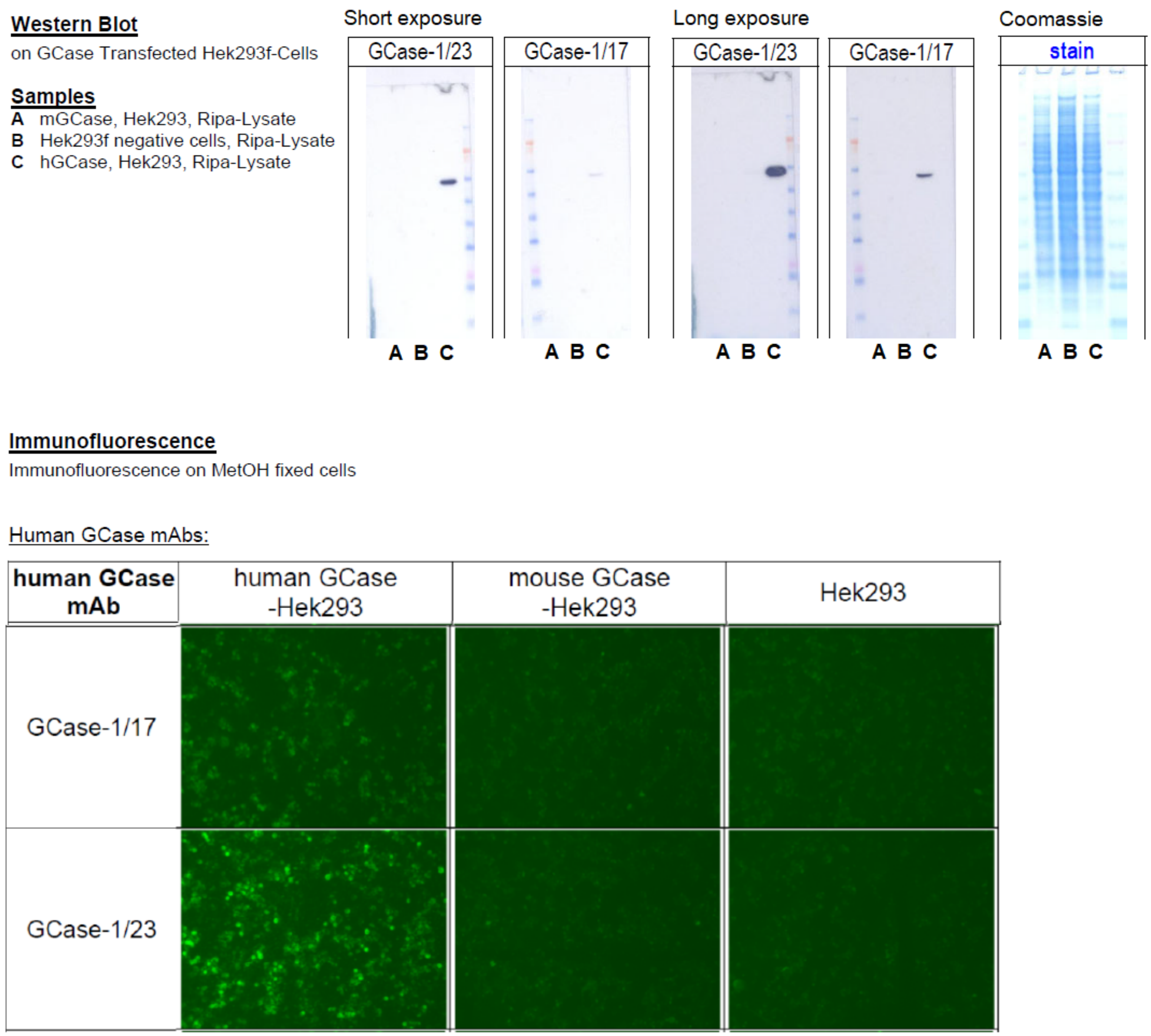

Figure S1. Characterization of hGCase-1/17 and hGCase-1/23 hybridoma clones

Hybridoma clones hGCase-1/17 and hGCase-1/23 were derived from mice immunized with imiglucerase. HGCase-1/23 demonstrated stronger potency than hGCase-1/17 towards hGCase overexpressed in HEK293-F cells in western blotting and in immunofluorescence assays. Neither of the two antibodies cross-reacted with mouse β-glucocerebrosidase (mGCase) overexpressed in HEK293-F cells, demonstrating their species specificity toward hGCase.

bioRxiv preprint doi: https://doi.org/10.1101/2023.09.14.557851; this version posted September 15, 2023. The copyright holder for this preprint (which was not certified by peer review) is the author/funder. This article is a US Government work. It is not subject to copyright under 17 USC 105 and is also made available for use under a CC0 license.

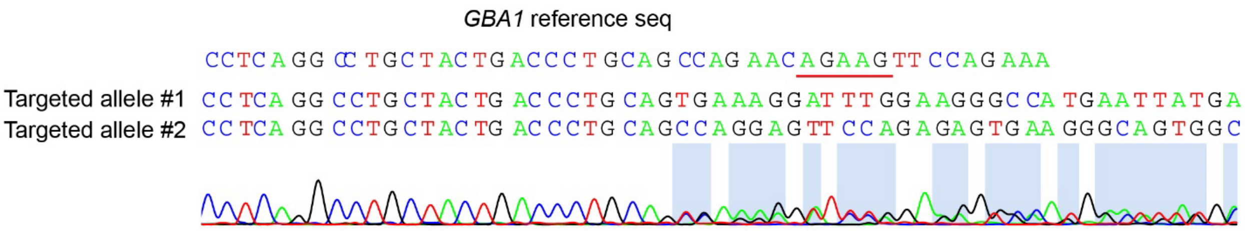

Figure S2. Frameshift indels in the two *GBA1* alleles targeted by ZFN but lacking resistance cassette integration.

Sanger sequencing *GBA1* alleles without resistance cassette integration showed double peaks in the chromatogram indicating the two *GBA1* alleles were targeted by ZFN as well. Parsing the double peak chromatogram using Poly Peak Parser revealed frameshift indels in both alleles.

bioRxiv preprint doi: https://doi.org/10.1101/2023.09.14.557851; this version posted September 15, 2023. The copyright holder for this preprint (which was not certified by peer review) is the author/funder. This article is a US Government work. It is not subject to copyright under 17 USC 105 and is also made available for use under a CC0 license.

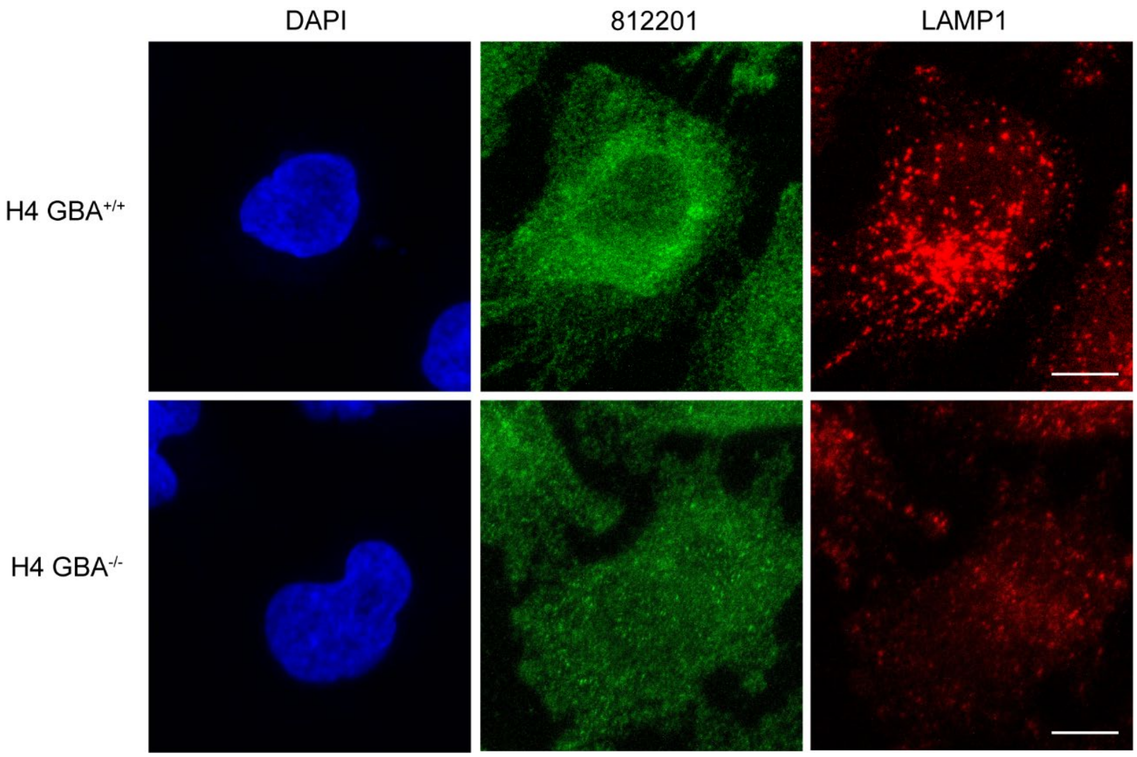

Figure S3. No specific immunostaining of hGCase in H4 cells with hGCase antibody 812201.

GBA1+/+ H4 cells were fixed with 4% PFA, permeabilized with 0.05% Saponin, and stained with hGCase antibody 812201, together with LAMP1 antibody. The staining pattern was diffusive showing no localization in lysosomes marked with LAMP1. Scale bar: 10 µm.
